# Widespread and increasing near-bottom hypoxia in the coastal ocean off the United States Pacific Northwest

**DOI:** 10.1038/s41598-024-54476-0

**Published:** 2024-02-15

**Authors:** John A. Barth, Stephen D. Pierce, Brendan R. Carter, Francis Chan, Anatoli Y. Erofeev, Jennifer L. Fisher, Richard A. Feely, Kym C. Jacobson, Aimee A. Keller, Cheryl A. Morgan, John E. Pohl, Leif K. Rasmuson, Victor Simon

**Affiliations:** 1 College of Earth, Ocean, and Atmospheric Sciences, Oregon State University, Corvallis, OR 97331 USA; 2 Pacific Marine Environmental Laboratory, National Oceanic and Atmospheric Administration, Seattle, WA 98115 USA; 3 Cooperative Institute for Climate, Ocean, and Ecosystem Studies, Seattle, WA 98105 USA; 4 Department of Integrative Biology, Oregon State University, Corvallis, OR 97331 USA; 5 Cooperative Institute for Marine Ecosystem and Resources Studies, Oregon State University, Newport, OR 97365 USA; 6 Northwest Fisheries Science Center, National Marine Fisheries Service, National Oceanic and Atmospheric Administration, Seattle, WA 98112 USA; 7 Oregon Department of Fish and Wildlife, Newport, OR 97365 USA

**Keywords:** Physical oceanography, Marine chemistry

## Abstract

The 2021 summer upwelling season off the United States Pacific Northwest coast was unusually strong leading to widespread near-bottom, low-oxygen waters. During summer 2021, an unprecedented number of ship- and underwater glider-based measurements of dissolved oxygen were made in this region. Near-bottom hypoxia, that is dissolved oxygen less than 61 µmol kg^−1^ and harmful to marine animals, was observed over nearly half of the continental shelf inshore of the 200-m isobath, covering 15,500 square kilometers. A mid-shelf ribbon with near-bottom, dissolved oxygen less than 50 µmol kg^−1^ extended for 450 km off north-central Oregon and Washington. Spatial patterns in near-bottom oxygen are related to the continental shelf width and other features of the region. Maps of near-bottom oxygen since 1950 show a consistent trend toward lower oxygen levels over time. The fraction of near-bottom water inshore of the 200-m isobath that is hypoxic on average during the summer upwelling season increases over time from nearly absent (2%) in 1950–1980, to 24% in 2009–2018, compared with 56% during the anomalously strong upwelling conditions in 2021. Widespread and increasing near-bottom hypoxia is consistent with increased upwelling-favorable wind forcing under climate change.

## Introduction

Coastal waters off the United States Pacific Northwest of North America are home to thriving marine ecosystems and coastal economies based on sustainable marine fisheries. These coastal waters are part of the northern California Current, a classic oceanic eastern boundary current where summertime, wind-driven coastal upwelling brings subsurface water upward near the coast. This water is high in nutrients and fuels a productive marine food web, one that includes multi-million dollar fisheries for bottom and near-bottom species of shellfish and groundfish like Dungeness crab, Petrale sole and Lingcod. In recent years, a number of threats to the continued sustainable marine harvest and concomitant coastal economies have been identified, including ocean warming^1^, low-oxygen^2^, ocean acidification^3,4^ and the occurrence of harmful algal blooms^5^. More information is needed about the spatial distribution and severity of these stressors so that threats to the marine ecosystem and coastal economies can be mitigated.

Upwelled waters off the United States Pacific Northwest are also low in oxygen, as the deep (~ 150–200 m) source waters for the upwelling have been isolated from the atmosphere for decades and have lost oxygen via respiration of organic matter along their journey from the northwest Pacific to the northeast Pacific off the United States Pacific Northwest coast. The upwelled water is low in oxygen, but still has enough to stay above the hypoxic threshold of < 61 µmol kg^−1^ (1.4 mL L^−1^) and shown to be harmful to many marine organisms^6^. These upwelling source waters are declining in oxygen^7^, consistent with a global decrease in oxygen of subsurface ocean waters under the effects of climate change^8^. Dissolved oxygen (DO) in near-bottom coastal waters is further decreased to below the hypoxic threshold by respiration of falling organic matter and in seafloor processes over the shelf. The effects of global deoxygenation and shelf processes are adversely impacting oxygen levels in the world’s other productive eastern boundary current regions: the Humboldt, Benguela and Canary Current Systems^8^.

The amount of upwelling and water-column primary production via phytoplankton growth are influenced by the amount of summertime, southward, upwelling-favorable winds in the northern California Current. It is hypothesized that these summertime winds over oceanic eastern boundary currents will increase under global climate change due to differential warming of the adjacent lands and coastal waters^9^. We report on widespread, near-bottom hypoxia off the United States Pacific Northwest during the summer of 2021. Understanding how susceptibility to upwelling-driven hypoxia varies regionally can inform management and policy decisions to improve the chances for continued sustainable use of these productive coastal waters under increasing human use and climate change.

## Results

### Summer 2021 spatial distribution of hypoxia

**Figure 1:**
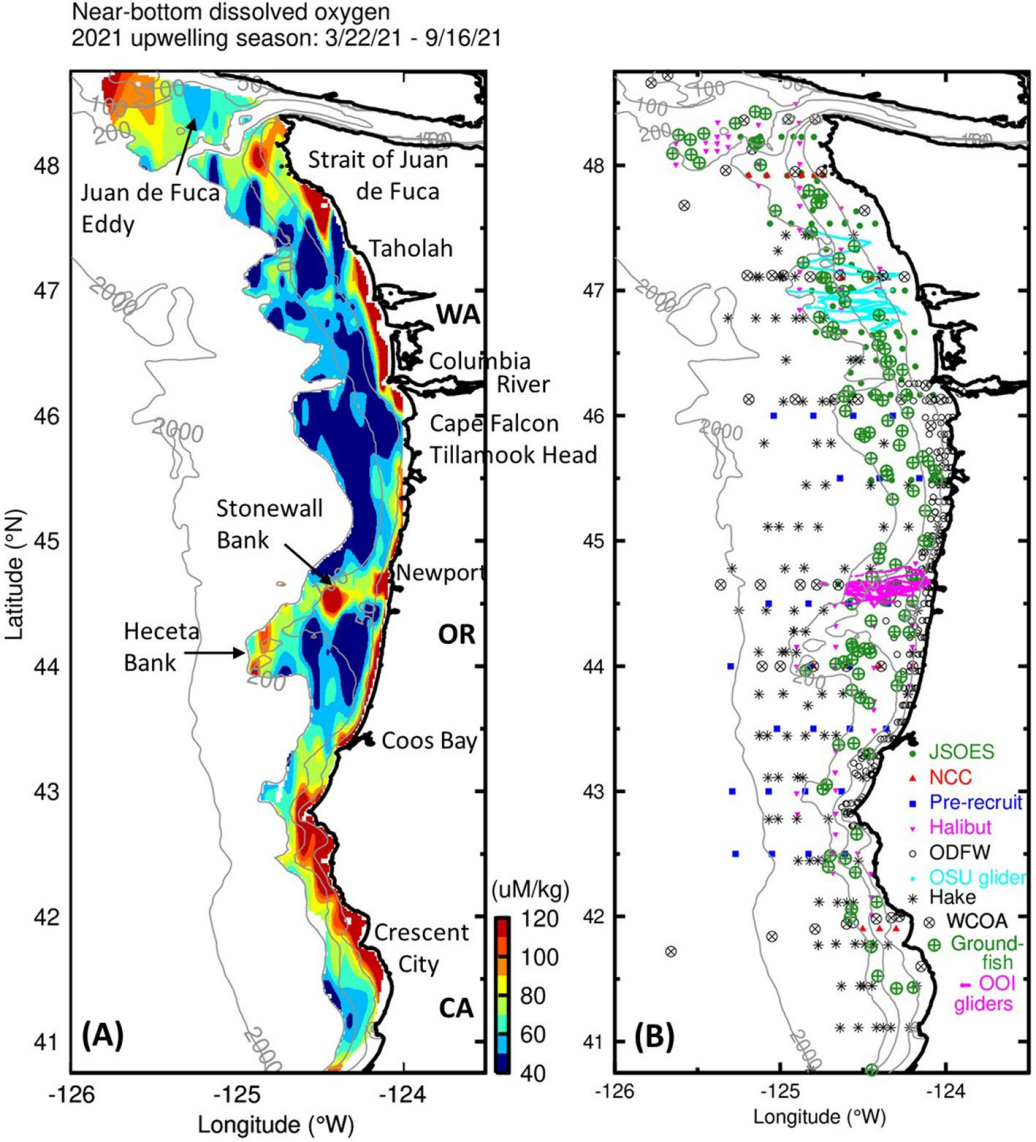
Maps of dissolved oxygen and sample locations during summer 2021. (**A**) Near-bottom dissolved oxygen in µmol kg^−1^; the blue-cyan transition at 61 µmol kg^−1^ denotes the hypoxia threshold. (**B**) Sample locations color-coded by program. Bottom depth in m; the 200-m isobath marks the edge of the continental shelf. Maps created with the Gri scientific graph language (Version 2.12.23, https://gri.sourceforge.net/index.php ).

**Figure 2:**
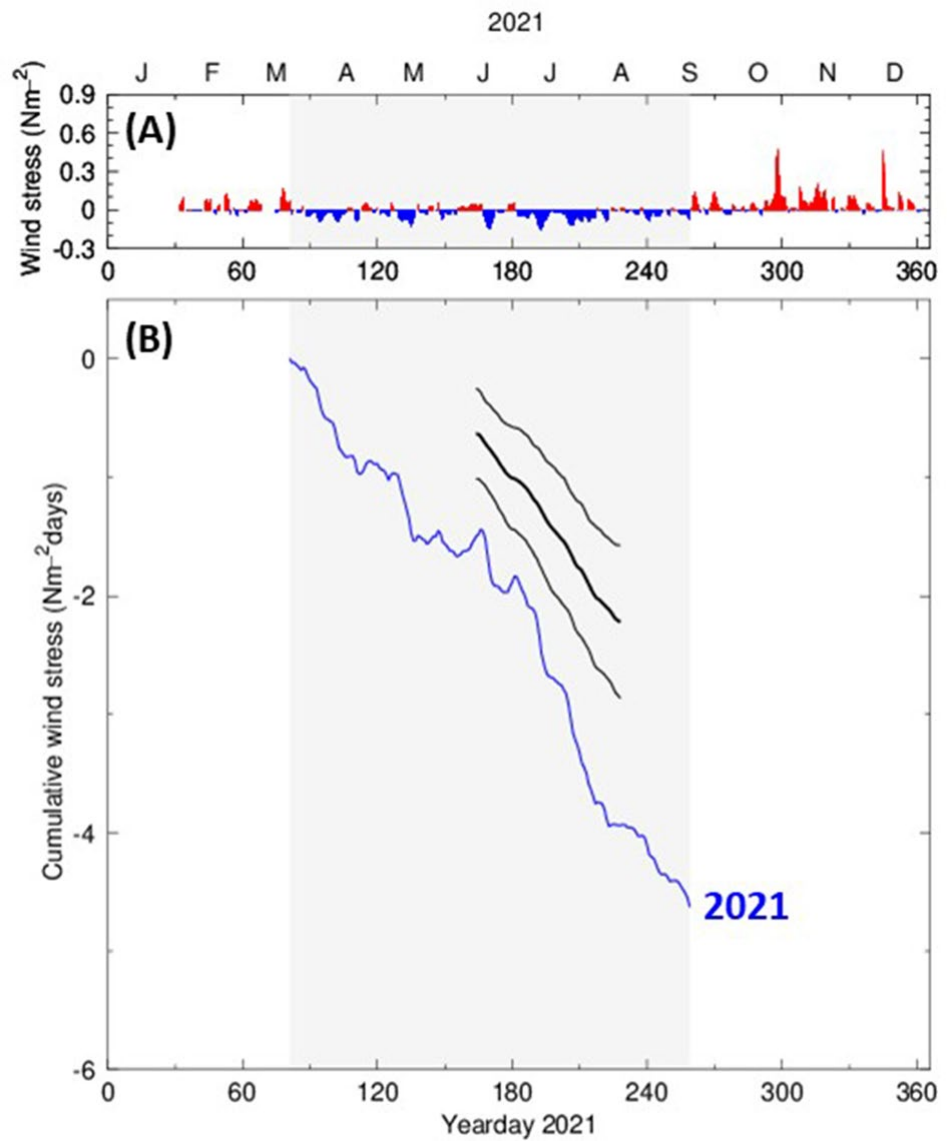
Winds during 2021. (**A**) North–south wind stress from Newport, OR; upwelling(downwelling)-favorable, south(north)ward wind is shaded blue(red); gray shaded region denotes the summer upwelling season. (**B**) Cumulative wind stress during 2021 (blue curve) compared with data from 1985 to 2022 (mean is thick curve; ± one standard deviation denoted by thin curves).

During summer 2021 there was an unprecedented number of measurements of oceanographic parameters in United States Pacific Northwest waters from a variety of ship-based and autonomous vehicles (Fig. 1). There were 4–10 times as many ship-based measurements compared with previous years and over 10,000 additional glider profiles. To map near-bottom DO, we used measurements during the 2021 upwelling season. The upwelling season is defined as starting from the spring transition when winds turn to predominantly upwelling-favorable (southward) usually during March to April^10^. The upwelling season ends at the fall transition when winds turn to predominantly downwelling-favorable (northward)^11^. The timing and duration of the summertime upwelling season directly influences marine ecosystems in the northern California Current^12^. For 2021, the upwelling season spans from March 22 to September 16, a total of 189 days (Fig. 2), compared with an average upwelling season length of 155 ± 28 days based on data from 1985 to 2021.

By combining ship- and glider-based observations from multiple projects, we mapped the upwelling-season average, near-bottom (< 10 m above the bottom) DO along nearly 900 km of the United States Pacific Northwest coast from 40.75 to 48.75°N (Fig. 1). The observations span from the inner shelf in water depth of about 10 m or less, out past the continental shelf break (~ 200 m). We plot the data inshore of the continental shelf break to focus on the water that has been upwelled on to the continental shelf from offshore and that is subject to physical and biological processes on the continental shelf.

Hypoxia was widespread across the shelf region of the northern California Current during summer 2021 (Fig. 1). Water near the coast over the inner shelf, where water depths are less than 25–50 m, was generally high in oxygen due to mixing by wind-driven currents and waves, and by increased contact with the atmosphere. Farther offshore, 48% of the continental shelf inshore of the 200-m isobath was hypoxic, an area of 15,500 km^2^. This total area is about the same size as Oregon’s Willamette Valley, stretching from Portland to Eugene (14,900 km^2^), or the Seattle metropolitan area (15,209 km^2^), slightly smaller than the US state of Connecticut (14,357 km^2^) or roughly half the size of Belgium (30,528 km^2^). This estimate for 2021 is similar to a previous estimate of near-bottom hypoxia in United States Pacific Northwest waters inferred from non-concurrent observations (Peterson et al., 2013). The near-bottom hypoxic zone in United States Pacific Northwest coastal waters is similar in size to the hypoxic zone associated with the Mississippi River outfall (5-year average for 2015–2019 of 15,000 km^2^)^13^.

While hypoxia is regional in scope, distinct areas of higher or lower DO are evident. We report on these from north to south, rather than by their relative intensities or size.

### High-oxygen regions

A region of relatively high oxygen water is the shelf region to the south of the Strait of Juan de Fuca, extending down to about 47.7°N. These high oxygen concentrations are likely caused by enhanced circulation and mixing from the strong tidal and subtidal currents associated with the Strait’s entrance. Summertime, subtidal mean velocities are to the south on the northern Washington shelf^14^, thus capable of carrying high-oxygen waters associated with the mouth of the Strait of Juan de Fuca southward into the northern Washington shelf region.

Two small regions of higher near-bottom DO are found off central Oregon over Stonewall (44.5° N, 124.4° W; rising to within 7 m of the sea surface on the ~ 70-m deep mid shelf) and Heceta (44.0–44.5° N, 124.75° W; rising to within 46 m of the sea surface on the 150–200 m deep outer shelf) Banks. The relatively high, near-bottom oxygen over Stonewall Bank was previously described using glider data and attributed to enhanced mixing due to interaction of coastal currents with Stonewall Bank^15^. This same process is likely responsible for the relatively high, near-bottom DO over Heceta Bank (~ 124.75° W).

The shelf south of Coos Bay, OR (43.5° N) also has high average near-bottom oxygen concentrations, likely associated with the substantial narrowing of the continental shelf in that region, that reduces the opportunity for sedimentary respiration. Near-bottom hypoxia was generally only found in the previous 1998–2012 surveys north of 43.5° N where the continental shelf widened^16^.

### Low-oxygen regions

Low-oxygen, near-bottom water is present in the region of the Juan de Fuca Eddy (center ~ 48.3° N, 125.3° W) as observed previously^17^. The near-bottom water in this region has been shown to originate in deeper waters offshore and is upwelled to beneath the Juan de Fuca Eddy by the favorable connection of the deep entrance to the Strait of Juan de Fuca^18^. DO concentrations are driven lower than found in the upwelling source waters via decay of organic matter retained in the eddy region^18,19^.

Much of the relatively wide Washington continental shelf between the Columbia River (46.25° N) and 47.7° N has hypoxic, near-bottom water. Lowest values (< 50 µmol kg^−1^) are found between Taholah (47.35°N) and 47.7°N. These low DO values reach all the way to the coast near Taholah, the location where, in 2006, another year of extensive near-bottom hypoxia off the United States Pacific Northwest^16,20,21^, significant fish die offs were found on beaches near the Quinault Indian Nation^22^. The low-oxygen, near-bottom water on the Washington shelf and the presence of a north–south gradient along the coast is consistent with observations during 2003–2006^21^, in 2009^23^, and in model simulations for this region run for 2005–2007^19^.

The largest regions of near-bottom hypoxia with the lowest levels (< 50 µmol kg^−1^) occur off the north-central Oregon and Washington coasts, and take the form of a “ribbon” extending along the coast in water depths of about 75–200 m. The mid continental shelf ribbon separates higher oxygen waters closer to shore from offshore waters that are low in oxygen, but not hypoxic because they have not experienced respiration of organic matter on the shelf. One region of this hypoxic beltway with near-bottom, DO levels less than 50 µmol kg^−1^ extends about 220 km from north of the Columbia River (46.7° N) to Newport (44.6° N), OR. Another mid-shelf region with low-oxygen (< 50 µmol kg^−1^) extends about 90 km in the alongshore direction over the Heceta Bank complex (44–44.75° N) and to the south. The Heceta Bank complex includes Heceta and Stonewall Banks and all the area inshore to the coast. This low-oxygen region extends nearly continuously across the bank inshore of the 200-m isobath, but is punctuated by higher-oxygen regions associated with Stonewall Bank as previously noted. Note that values of near-bottom DO along the historic Newport Hydrographic Line (44.6°N) reported here are higher than the range of 0.8–1.2 mL L^−1^ (35–50 µmol kg^−1^) reported for this same location using underwater glider data from 2006 to 2012 (*15*), but approximately within the same range as that found for the same region during the summer of 2007^24^. This is because the average reported here is for all summertime upwelling season data, including measurement times when winds were relaxed or downwelling-favorable early in the upwelling season especially as measured by the OOI gliders, rather than the average determined only from those sections that showed hypoxia^15^.

**Figure 3:**
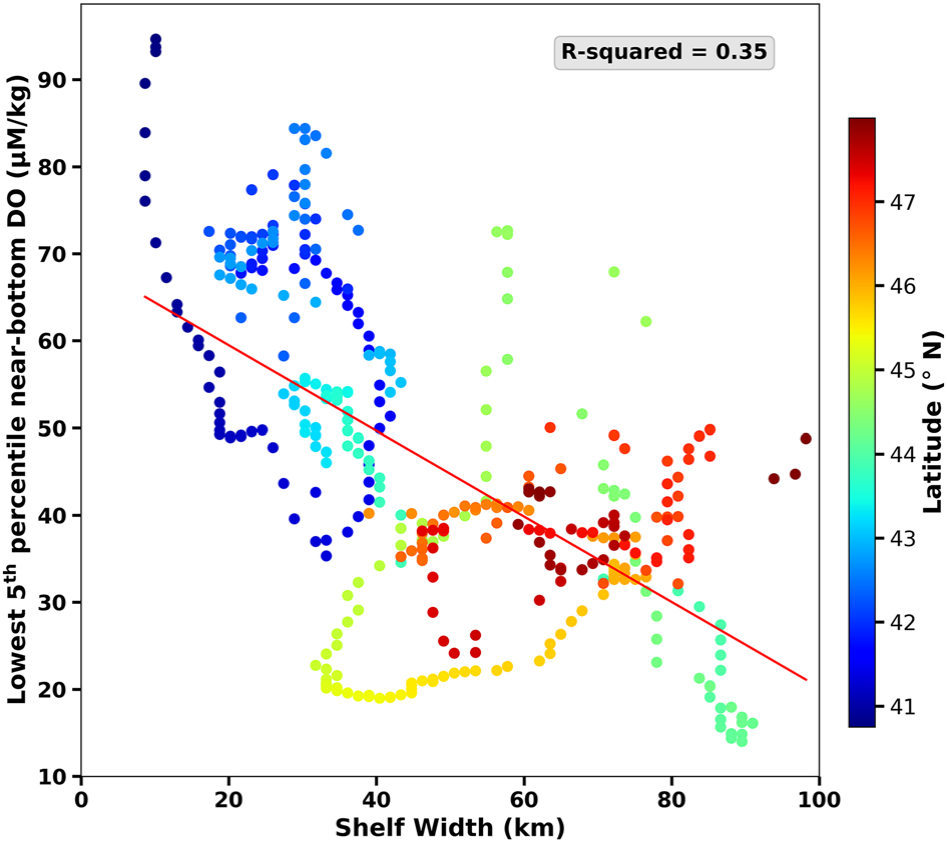
The lowest fifth percentile of near-bottom dissolved oxygen as a function of continental shelf width, color-coded by latitude. Individual data points represent fifth-percentile values computed between the coast and the 200-m isobath every 2 km from 40.75 to 48°N.

To test the relationship between the amount of hypoxic, near-bottom water and continental shelf width, we plot the lowest fifth percentile near-bottom DO versus shelf width (Fig. 3). This relationship is calculated from gridded oxygen data and computed along a line of constant latitude every 2 km along the coast. We exclude the region north of 48N since that area is complicated by the Strait of Juan de Fuca Canyon and the coastline veers from north–south. Previous work has suggested that wider, shallow shelves have greater sediment oxygen demand leading to more hypoxia^19^. We found DO is highest for narrow shelves (< 30 km) and lowest for wide shelf widths (40–60 km). However, there are exceptions, with regions where the shelf is narrow and oxygen is low—this is from the region south of Crescent City, CA—and regions where the shelf is wide and oxygen concentrations are not hypoxic, e.g., on the northern Washington shelf south of the entrance to the Strait of Juan de Fuca. There is a significant relationship between the two, with an r^2^ of 0.35.

Overall, nearly half of the continental shelf along approximately 900 km off the United States Pacific Northwest coast from 40.75 to 48.75°N was hypoxic on average during the 2021 summer upwelling season. A mid-shelf ribbon with near-bottom, DO less than 50 µmol kg^−1^ extended for 450 km off north-central Oregon and Washington. To estimate the volume of hypoxic water on the shelf in this region, we use estimates of 25 m for the thickness of the near-bottom hypoxic layer^2,15^. Using 10-m as a lower bound and 25-m as an upper bound, the volume of the hypoxic region varies between 645 and 1612 cubic kilometers. This is 5–10 times larger than the near-bottom, hypoxic volume associated with the Mississippi River outflow^13^.

### Mechanisms influencing near-bottom dissolved oxygen distributions

Waters near the coast, in depths less than about 25–50 m, are generally high in oxygen due to enhanced surface to bottom mixing in this region and relatively high contact of subsurface waters with the atmosphere. These regions of high oxygen can serve as refugia for near-bottom dwelling marine organisms during late-summer hypoxia. In contrast, low-oxygen water can reach very close to shore when active upwelling circulation brings near-bottom water shoreward^25^. On average during summer 2021, hypoxic waters reach close to shore off Taholah, WA (43.75° N) and also from Cape Falcon (45.75° N) to Tillamook Head (45.95° N), Oregon (Fig. 1). The mechanisms responsible for the exposure of these particular inner-shelf regions to low oxygen are uncertain and deserve further research.

The hypoxic region offshore and to the south of the Columbia River (46.25° N) can be interpreted in terms of the near-bottom respiration of larger- or more intense-than-average phytoplankton blooms associated with the Columbia River plume. Mixing at the bottom of the shallow plume draws relatively high-nutrient oceanic water up into the well-lit euphotic zone in the strongly stratified plume waters^26^. This leads to enhanced phytoplankton production that sinks and enhances respiratory draw down of oxygen near the sea floor. Summertime currents in this region are to the south^26^, pushing the low-oxygen, near-bottom hypoxic region southward. This low-oxygen, near-bottom water may be drawn into the lower Columbia River estuary during coastal upwelling, driving observed low-oxygen in that region^27^.

The low-oxygen region extending about 90-km in the alongshore direction over the Heceta Bank complex and to the south (43.5–44.5° N) results from a flow-topography interaction as the strong, southward, mid-shelf coastal upwelling jet encounters the shallow submarine bank^28^. The mid-shelf, southward coastal upwelling jet is pushed offshore around the outside of the Bank, because geophysical flows in a rotating, stratified fluid tend to follow isobaths rather than flow over an obstacle. This leaves a region of low flow velocity over the Bank that reduces advective loss of waters, and the material they contain, offshore. This combines with increased upwelling to maintain cyclostrophic balance, to create a region with increased phytoplankton production^28^. This enhanced organic matter can die and sink to the seafloor, driving near-bottom hypoxia over the Bank. In situ^28^ and satellite^29^ measurements confirm that summertime mean surface chlorophyll is high on the inshore part of the Heceta Bank complex, consistent with the idea of enhanced vertical export and respiration leading to near-bottom hypoxia in this region. We note that this relationship between low near-bottom DO and high near-surface, satellite-derived chlorophyll^29^ holds true for much of the Washington shelf, with the exceptions noted here south of the Strait of Juan de Fuca and in the inner shelf. However, there are other regions where this relationship breaks down, notably over the narrow shelf regions south of Cape Blanco, Oregon, noted here where more efficient shelf flushing is important.

### Maps of near-bottom hypoxia over time

**Figure 4:**
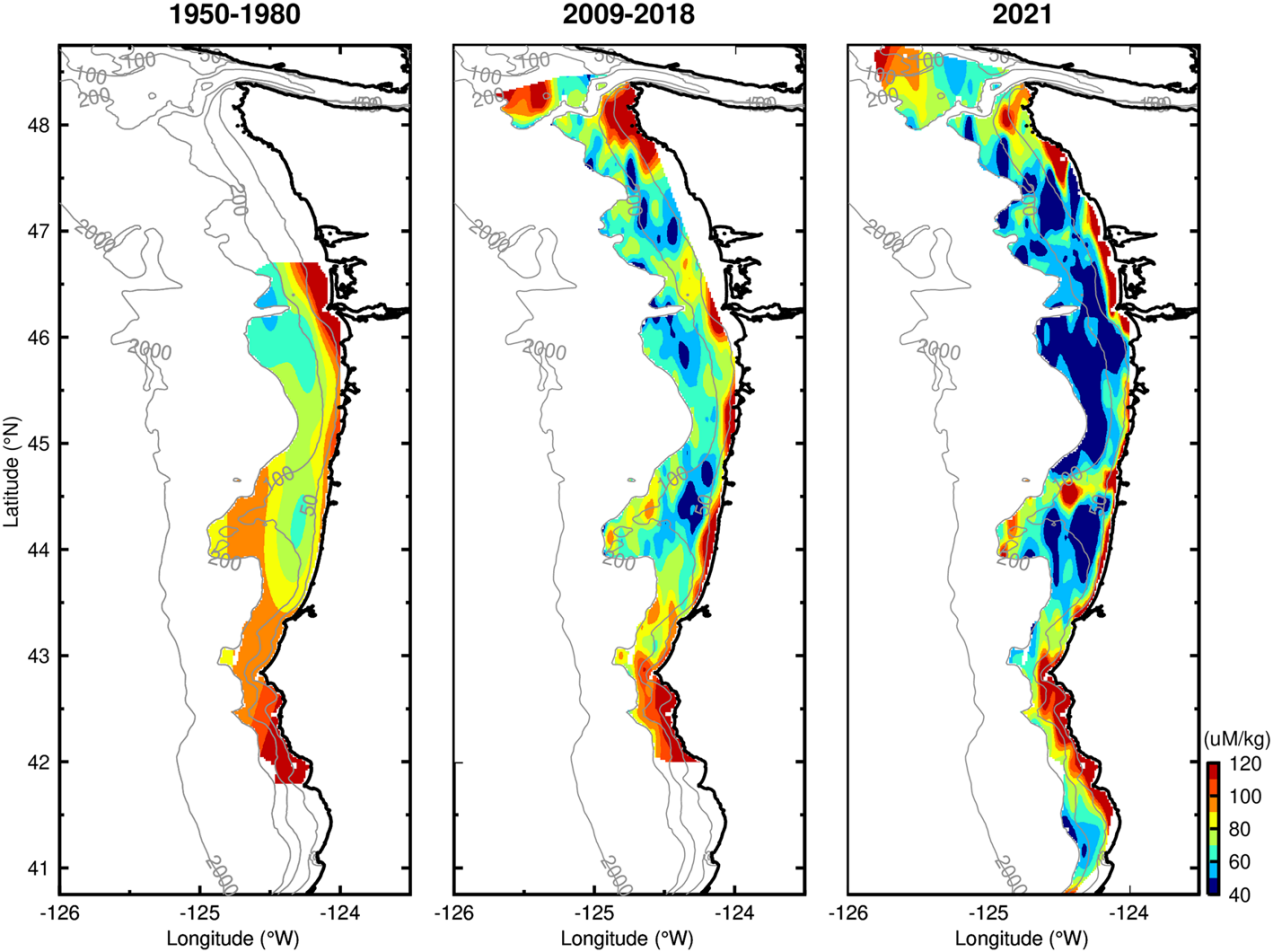
Maps of near-bottom dissolved oxygen over time. Data from 1950 to 1980 are from the World Ocean Database^32^, data from 2009–2018 are from the NOAA Groundfish survey, and data from 2021 are from this study. Maps created with the Gri scientific graph language (Version 2.12.23, https://gri.sourceforge.net/index.php).

Dissolved oxygen levels in near-bottom continental shelf waters off the United States Pacific Northwest have been decreasing over the last half century^7^. To place the 2021 near-bottom dissolved oxygen maps in context, we present additional maps computed from summer upwelling season data collected during 1950–1980 and from the NOAA groundfish surveys from 2009 to 2018 (Fig. 4). Two results are immediately apparent. First, the areas of both high and low, near-bottom dissolved oxygen appear in similar locations across decades, lending strength to the interpretation that these areas are determined by oceanographic processes. Second, near-bottom, dissolved oxygen levels are decreasing over time across continental shelf waters off the United States Pacific Northwest.

All three maps confirm high oxygen over the inner shelf in water depths less than 25–50 m depth. High near-bottom dissolved oxygen is found in all three maps south of Coos Bay, OR (43.5°N), where the continental shelf is narrower. The relatively high, near-bottom DO region associated with outer Heceta Bank (44.0–44.5° N, 124.5–125° W) is visible in the two most recent maps and is even hinted at in the lower spatial resolution data from 1950 to 1980. Farther north, the 2009–2018 maps confirm the 2021 presence of relatively high oxygen water to the south of the Strait of Juan de Fuca, extending down to about 47.7° N.

For the common overlap area—sampled in all maps—from the 1950s to 2021, low, near-bottom dissolved oxygen is found south of the Columbia River plume in the wide shelf region near 46° N. The low-oxygen zone associated with the Heceta Bank complex is also present across decades. Farther north, the 2009–2018 maps confirm the presence of low-oxygen, near-bottom waters in the Juan de Fuca Eddy (center ~ 48.3° N, 125.3° W), with relatively little change over time. The 2009–2018 map also corroborates the presence of low, near-bottom dissolved oxygen over the wide continental shelf off Washington. Low-oxygen, near-bottom waters extend all the way to the coast near Taholah, WA (47.35° N) in the 2009–2018 map.

The fraction of near-bottom water in the common overlap area inshore of the 200-m isobath that is hypoxic (DO < 61 µmol kg^−1^) on average during the summer upwelling season increases over time from nearly absent (2%) in 1950–1980, to 24% in 2009–2018, compared with 56% during the anomalously strong upwelling conditions in 2021. The corresponding increasing areas of near-bottom hypoxia in the common overlap area over time are 367, 4834, and 11,333 km^2^ from 1950 to 1980, 2009–2018 and 2021, respectively. In the same common overlap area, the average near-bottom DO inshore of the 200-m isobath decreases from 87, to 78 ± 11, to 69 µmol kg^−1^ from 1950 to 1980, 2009–2018 and 2021, respectively. The standard deviation among the individual groundfish surveys from 2009–2018 serve as an estimate for year-to-year variability in average near-bottom DO in this region. The decrease of 9 µmol kg^−1^ between the 1950–1980 and 2009–2018 average near-bottom shelf DO represents a 10% decrease over 50 years (− 0.18 µmol kg^−1^ y^−1^). This is less than other recent estimates of the decline in near-bottom DO from single locations, e.g., a 35% decline in 50 years, equivalent to -0.7 ± 0.2 µmol kg^−1^ y^−1^ at 50 m at NH-5 (60-m water depth, 44.652° N, 124.17° W)^7^, likely because this new shelf-wide trend averages over areas with both higher and lower decreasing trends in near-bottom DO. Note that we do not calculate a decreasing trend using the anomalous 2021 measurements. The decline in near-bottom DO extends across much of the continental shelf off the United States Pacific Northwest Coast, thus not limited to single points or single cross-shelf lines. Perhaps most striking is the extent of near-bottom waters with the lowest values of oxygen (< 50 µmol kg^−1^, dark blue) in 2021 compared with the previous decade.

## Discussion

**Figure 5:**
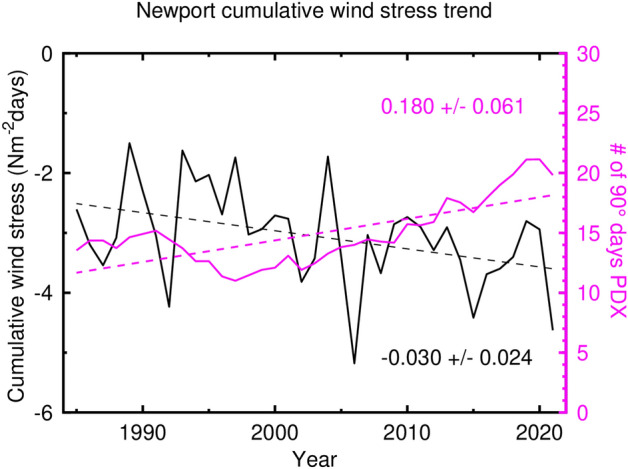
Trends in wind forcing and summertime temperatures. Summertime cumulative, alongshore, wind stress—negative is more upwelling-favorable—and number of 90°F days in Portland, Oregon, the latter smoothed with a 10-year running mean filter (in 2021 there were twenty-four 90° days).

Why was hypoxia so widespread in 2021? While the relevant physical and biological processes involved are many and varied, we focus on how the amount of hypoxia is related to the total amount of wind-driven, summertime upwelling. The cumulative upwelling wind stress for 2021 was greater by more than two standard deviations when compared to the average for the last 36 years (Fig. 2). Cumulative upwelling during summer 2021 was the second highest amount of upwelling in the last 35 years (Fig. 5). The highest year on record was 2006 when widespread hypoxia was noted^16,21^ and anoxia was recorded for the first time in the instrumental record off central Oregon^20^. Cumulative summertime upwelling is increasing at a rate of 0.030 ± 0.024 N m^−2^ days per year. While 2021 was an extreme but not uncommon year of cumulative summertime upwelling—one of three large upwelling years in the last 15 years—this amount of summertime upwelling could become routine by the year 2050 under continued climate change. An increase in alongshore, upwelling favorable winds is consistent with the hypothesis that under global climate change the land-sea temperature differences will increase leading to an increased on-offshore pressure gradient and a concomitant increase in geostrophic, upwelling-favorable winds^9^. The number of 90°F days at the Portland, OR, airport is increasing over the last 25 years at a rate of 0.180 ± 0.061 days per year (Fig. 5). By extension then, we might expect to see more widespread hypoxia in the northern California Current as climate change continues^30^. We were unable to find a statistical relationship between either the spatial extent of near-bottom hypoxia or the average near-bottom DO and cumulative upwelling intensity. This is because the record of shelf-wide, near-bottom measurements across the entire coastal ocean off the United States Pacific Northwest collected in one upwelling season is too short—shelf-wide measurements begin with the 2009 NOAA groundfish surveys—and is confounded by the anomalous marine heat wave years of 2014–2017^1^. Sustained measurements will be needed to assess the extent of hypoxia along this critical shelf area and how it varies over time in response to natural and anthropogenic forcing.

That many of the low- and relatively high-oxygen features brought into sharp focus by the unprecedented 2021 observations were apparent in data from previous decades suggests that they are spatially persistent. Their location and size are associated with oceanographic processes on the United States Pacific Northwest continental shelf. These processes lead to both relatively high (inner shelf air-sea exchange and mixing; low retention on narrow continental shelves; mixing by tidal currents; mixing by flow over rough topography) and low (high retention on wide continental shelves; flow-topography interaction leading to relatively quiescent regions; sinking and decay of river-plume enhanced primary production) near-bottom DO concentrations.

The decrease of dissolved oxygen content of near-bottom waters off the United States Pacific Northwest, and concomitant increase in the area of near-bottom hypoxia, can be due to both changes in remote and local processes. Remote processes include changes in the dissolved oxygen content of upwelling source waters delivered to the edge of the continental shelf by basin-scale circulation. Subsurface dissolved oxygen levels, especially those at the 150–200 m depths of the source waters for upwelling off the United States Pacific Northwest coast, are decreasing globally due to a combination of climate change effects^8^. The increase we observe in the area of near-bottom hypoxia is consistent with the decrease of dissolved oxygen content observed just offshore of the shelf at the upwelling source water depths^7^. Subsurface dissolved oxygen concentrations at upwelling source water depths can be influenced by decadal variability in the Northeast Pacific, in particular the Pacific Decadal Oscillation (PDO) influencing the southern California Current^31^. The sampling periods of the three maps shown in Fig. 4 are primarily from years of negative PDO anomaly, minimizing the influence of decadal variability. Local processes could include changes in shelf primary production, stratification, and retention. Future work will address the relative contributions of these remote and local processes to increased near-bottom hypoxia off the United States Pacific Northwest coast.

Lastly, there are additional metrics of near-bottom hypoxia that deserve attention in addition to the summer-season average considered here. For example, the temporal duration of hypoxia within the upwelling season and how that varies with space, as the exposure time of organisms to hypoxia is important. This can be addressed at some locations by time series of near-bottom measurements of dissolved oxygen from moored instruments or underwater gliders. The use of verified high-resolution coupled ocean circulation and biogeochemical numerical models will also be valuable [e.g.^19^].

## Methods

We collected data from online data repositories and from lead investigators of various at-sea observational programs**.** These include the World Ocean Data Base^32^, ship surveys conducted by the U. S. National Oceanic and Atmospheric Administration (NOAA): Northern California Current (NCC); Pre-recruit; Juvenile Salmon and Ocean Ecosystem Survey (JSOES); Hake; Groundfish; and West Coast Ocean Acidification (WCOA), and the International Pacific Halibut Commission (IPHC). The Oregon Department of Fish and Wildlife (ODFW) conducted a survey over the inner shelf. Underwater glider measurements were made by Oregon State University (OSU) and the U.S. National Science Foundation-funded Ocean Observatories Initiative (OOI) (Table 1).

**Table 1:** Dissolved oxygen data sets during the March 22 to September 16, 2021, upwelling season.

Sampling Program	Data source
NOAA Juvenile Salmon and Ocean Ecosystem Survey; Northern California Current; Pre-recruit	https://www.fisheries.noaa.gov/west-coast/science-data/research-surveys-pacific-northwest#what-we-do
NOAA Hake	https://www.fisheries.noaa.gov/west-coast/science-data/joint-us-canada-integrated-ecosystem-and-pacific-hake-acoustic-trawl-survey
NOAA Groundfish	https://www.fisheries.noaa.gov/west-coast/science-data/us-west-coast-groundfish-bottom-trawl-survey
NOAA West Coast Ocean Acidification Survey	https://www.pmel.noaa.gov/co2/story/2021+West+Coast+Ocean+Acidification+Cruise
International Pacific Halibut Commission	https://iphc.int/datatest/data/water-column-profiler-data
Oregon Department of Fish and Wildlife	https://www.dfw.state.or.us/MRP/fisheries/
Oregon State University underwater gliders	http://gliderdac.ioos.org
Ocean Observatories Initiative underwater gliders	https://ooinet.oceanobservatories.org; Reference Designators CE05MOAS-GL311-12-CTDGVM000, CE05MOAS-GL319-10-CTDGVM000, CE05MOAS-GL320-8-CTDGVM000, CE05MOAS-GL326-9-CTDGVM000, CE05MOAS-GL326-10-CTDGVM000, CE05MOAS-GL382-9-CTDGVM000, CE05MOAS-GL386-13-CTDGVM000

*NOAA* National Oceanic and Atmospheric Administration.

Each at-sea measurement program applied their own data processing and quality control procedures. All relied on pre- and post-deployment factory and/or laboratory calibration of the dissolved oxygen (DO) sensors [e.g.^15^]. Some programs also took discrete, in situ water samples alongside the electronic sensors [e.g.^33^]. These samples were analyzed for their DO content via a Winkler titration, then compared with electronic sensor measurements of the same water. Any needed corrections were applied to the sensor data.

To define the 2021 upwelling season, north–south wind stress off central Oregon, used as a proxy for winds across United States Pacific Northwest waters, was computed from winds measured at NOAA’s Newport, Oregon, C-MAN (NWPO3) station^34^. The hourly data are low-pass filtered to remove diurnal variations. The spring and fall transitions are estimated from the alongshore wind stress record, using a CUSUM algorithm for change-point detection^35,36^. The significance (95%) of these two mean-shift change-points is confirmed using bootstrapping^37^.

**Figure 6:**
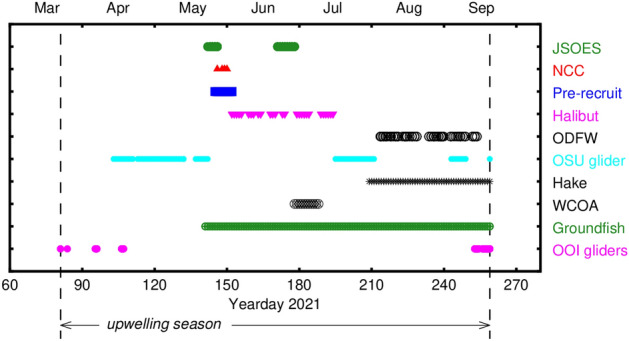
Time distribution of sampling of near-bottom dissolved oxygen during 2021. The dashed vertical lines indicate the duration of the 2021 summer upwelling season.

A total of 794 vertical profiles of oceanographic parameters were used to map near-bottom (< 10 m above the bottom) DO during the 2021 upwelling season as defined by the spring and fall transitions calculated from the Newport wind stress. This number of profiles is based on counting one profile for every multi-day to week underwater glider mission. If each glider vertical profile is counted, the total is 10,843 profiles, over one order of magnitude larger than the ship-based measurements alone. Ship- and glider-based measurements were distributed across the 2021 upwelling season with the ship-based measurements starting about 2 months after the 2021 spring transition (Fig. 6). Most ship schedules are made well in advance, often a year or more, so summer upwelling-season surveys are planned for mid-May or later to ensure that they occur after upwelling has commenced.

**Figure 7:**
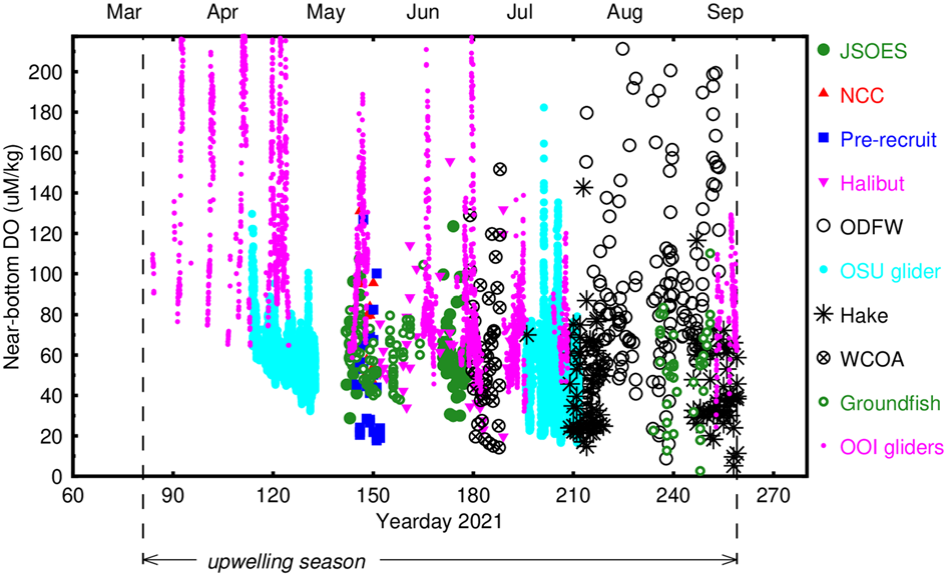
Near-bottom dissolved oxygen as a function of time during the 2021 summer upwelling season.

Near-bottom dissolved oxygen data from each survey as a function of time show the typical decrease of minimum values as the summer hypoxia season proceeds [e.g.^7,21,25^] (Fig. 7). High DO values are measured by the OOI gliders early in the upwelling season when winds were relaxed or downwelling-favorable early in the upwelling season, and by the ODFW survey that focused on very shallow (water depths of 50 m or less), inshore waters.

The near-bottom DO data are spatially interpolated to a rectangular grid spanning 40.75–48.75° N and 124–126° W with grid spacing of 0.013 degrees-longitude (~ 1 km) in the east–west direction and 0.018 degrees-latitude (~ 2 km) in the north–south direction. The grid is very high resolution to allow fine details of the spatial structure to be captured from the excellent data coverage. The near-bottom DO data are mapped using second-order thin plate smoothing splines^38^. Thin plate splines are flexible in how closely they adapt to fit local data points, according to a smoothing parameter. Optimizing the degree of smoothing is important. Here we optimize using the Generalized Cross Validation (GCV)^39^. The GCV measures the predictive error of the fitted surface by omitting each data point in turn and summing the square of the discrepancy of each data point from a surface fitted using all other data points. The smoothing parameter that yields the smallest GCV is chosen. Using thin plate splines which minimize the GCV is a method widely used when mapping data with sparse sampling, irregular distribution, and some degree of noise. Another advantage of the method is that a prior estimate of the spatial covariance structure is not required. We use the Linux spline package maintained by the Australian National University^40^.

## Data Availability

Wind data are available at https://www.ndbc.noaa.gov/station_page.php?station=nwpo3. The combined DO data set mapped to a regular latitude–longitude grid (40.75–48.75°N, 124–126°W with grid spacing of 0.013 degrees-longitude in the east–west direction and 0.018 degrees-latitude in the north–south direction) is available at https://oregonstate.box.com/s/hkkgeru08pcfiommj7f3wsfpqdy04qun. Individual data sets are listed in Table 1. Cumulative Upwelling Indices are available at https://www.nanoos.org/products/cui_oregon/cui.php. Number of 90°F days for the Portland International Airport, OR (station OR6751) are available at https://mesonet.agron.iastate.edu.
